# Efficacy of A Poly(MeOEGMA) Brush on the Prevention of *Escherichia coli* Biofilm Formation and Susceptibility

**DOI:** 10.3390/antibiotics9050216

**Published:** 2020-04-29

**Authors:** Patrícia Alves, Luciana Calheiros Gomes, Cesar Rodríguez-Emmenegger, Filipe José Mergulhão

**Affiliations:** 1 LEPABE—Laboratory for Process Engineering, Environment, Biotechnology and Energy, Faculty of Engineering, University of Porto, 4200-465 Porto, Portugal; 2 DWI—Leibniz Institute for Interactive Materials and Institute of Technical and Macromolecular Chemistry, RWTH Aachen University, Forckenbeckstraße 50, 52074 Aachen, Germany

**Keywords:** polymer brush, *Escherichia coli*, biofilm formation, antifouling properties, antibiotic susceptibility

## Abstract

Urinary tract infections are one of the most common hospital-acquired infections, and they are often associated with biofilm formation in indwelling medical devices such as catheters and stents. This study aims to investigate the antibiofilm performance of a polymer brush—poly[oligo(ethylene glycol) methyl ether methacrylate], poly(MeOEGMA)—and evaluate its effect on the antimicrobial susceptibility of *Escherichia coli* biofilms formed on that surface. Biofilms were formed in a parallel plate flow chamber (PPFC) for 24 h under the hydrodynamic conditions prevailing in urinary catheters and stents and challenged with ampicillin. Results obtained with the brush were compared to those obtained with two control surfaces, polydimethylsiloxane (PDMS) and glass. The polymer brush reduced by 57% the surface area covered by *E. coli* after 24 h, as well as the number of total adhered cells. The antibiotic treatment potentiated cell death and removal, and the total cell number was reduced by 88%. Biofilms adapted their architecture, and cell morphology changed to a more elongated form during that period. This work suggests that the poly(MeOEGMA) brush has potential to prevent bacterial adhesion in urinary tract devices like ureteral stents and catheters, as well as in eradicating biofilms developed in these biomedical devices.

## 1. Introduction

Bacterial biofilms are a severe problem in the pathogenesis of biomaterial-associated infections (BAI), leading to significant patient morbidity and mortality, and high costs to health services [1,2]. Over the past century, the use of medical devices, such as catheters, prosthetic joints, stents, artificial heart valves, pacemakers and other implants, has increased dramatically, which may result in BAI [3,4]. According to the American National Institutes of Health, the development and persistence of biofilms are responsible for 75% of all human bacterial infections [1]. Catheter-associated urinary tract infection (CAUTI) is one of the most common hospital-acquired infections [5,6] where *Escherichia coli* plays a key role due to its ability to adhere to biomaterials [7,8,9,10]. *E. coli* has also shown to persist and regrow after antibiotic exposure due to many factors, including reduced penetration of antibiotics in biofilms and/or cellular extrusion of drugs [7]. This may be due to the fact that biofilm cells produce a complex polymeric matrix, which protects the biofilm from antibiotic penetration and whose structure is strongly influenced by the substratum on which the biofilm forms [11,12] and by the bulk liquid phase which flows over the biofilm [13,14]. 

The high resistance of biofilms to conventional antibiotic therapy has driven research into the development of novel surfaces to decrease the attachment of microorganisms and consequent biofilm formation [15,16]. Polymer brushes have been developed to prevent the adsorption of biomolecules and the adhesion process by limiting the contact of substratum with living bacteria [17,18,19], which is particularly relevant in biomedical applications [20,21,22]. Furthermore, several polymer brushes based on 2-hydroxyethyl methacrylate (HEMA), oligo(ethylene glycol), 2-hydroxyethyl acrylamide (HEAA) [23], oxazolines [24], carboxybetaines, and N-(2-hydroxypropyl) methacrylamide (HPMA), including the one tested in this work (poly[oligo(ethylene glycol) methyl ether methacrylate], poly(MeOEGMA)), were shown to be resistant to the adsorption of compounds from human biological fluids such as blood plasma, urine, cerebrospinal fluid and saliva, as well as different cell lines and bacteria [17,20,25,26,27,28]. Moreover, a study using single-cell force spectroscopy demonstrated that antifouling polymer brushes can reduce the forces involved in bacterial attachment [29]. For instance, the force needed to detach *Yersinia pseudotuberculosis* was reduced over three orders of magnitude when comparing to Teflon or glass [29].

Besides the surface properties, the complex process of biofilm formation depends on multiple factors such as hydrodynamics and nutrient availability [30,31]. Several platforms for biofilm studies have been developed over the past decades [32]. Parallel plate flow cells (PPFCs) have been used for the study of biofilm formation under environmentally relevant hydrodynamic conditions, enabling a better understanding of the factors affecting the initial bacterial adhesion, which is the onset of biofilm development, as well as the online monitoring of biofilm development [14,33,34]. In recent work using a PPFC, two polymer brushes with well-known antifouling properties—poly(MeOEGMA) and poly(HPMA)—reduced the adhesion of *E. coli* after 30 min over a relevant range of shear stresses (0.005 to 0.056 Pa), including the one reported for urinary catheters (shear rate of 15/s) [22]. Shear stress values within this range are also found in some problematic areas of ureteric stents that are prone to bacterial accumulation and encrustation [35,36]. These polymer brushes also shown to be resistant to the adsorption of macromolecules from urine and serum, being promising surfaces to delay cell attachment in biomedical devices [22]. However, the mentioned investigation was performed using citrate buffer as a liquid medium to enable a more accurate thermodynamic analysis of the initial adhesion process. For these reasons, it is important to perform biofilm formation assays using a suitable medium that mimics the environment of interest since the fluid composition affects the behavior of pathogens involved in clinical infections [37,38]. Although many studies were performed aiming to better understand the bacterial attachment to different surfaces, few consider the importance of shear flow in bacteria-surface interactions [13,14,21,39,40]. 

The purpose of this work was to evaluate the antifouling performance of the poly(MeOEGMA) brush on *E. coli* biofilm formation and antimicrobial susceptibility. This polymer brush was selected due to its resistance to protein adsorption and bacterial adhesion (up to 90% when compared to glass) [22]. A well-described PPFC system [14] was used where synthetic urine was flown to mimic the shear rate typically found in urinary catheters and ureteric stents. The effect of the polymer brush on biofilm architecture was analyzed and compared with two control surfaces, polydimethylsiloxane (PDMS; a material commonly used for urinary catheters manufacture) and glass (surface used as a standard reference for adhesion studies). This work suggests that the poly(MeOEGMA) brush has the potential to reduce infections in urinary tract devices with high efficiency by reducing the biofilm growth and by making the biofilms formed in these biomedical devices more susceptible to the current antibiotic therapies.

## 2. Results

**Figure 1:**
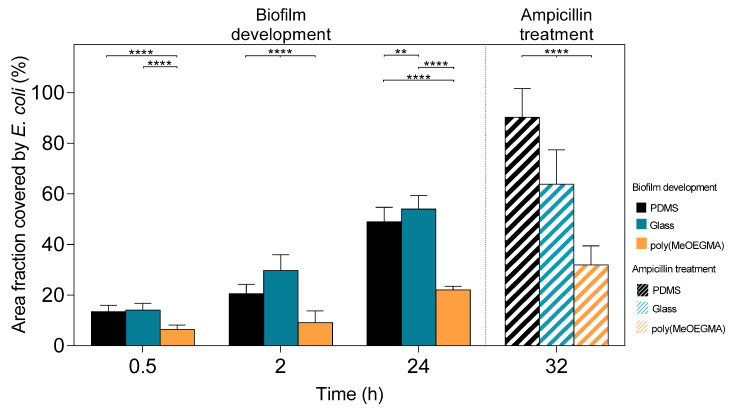
Surface area fraction covered during biofilm development (up to 24 h) and ampicillin treatment for an additional 8 h period (32 h) on polydimethylsiloxane (PDMS) (■ and 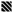, respectively), glass (■ and 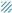, respectively), and poly(MeOEGMA) brush (■ and 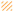, respectively). Standard deviations for three independent samples are presented. Statistical significance for all the surfaces at each time point was determined by one-way ANOVA followed by Tukey’s multiple-comparison test (**** for *p* < 0.0001, and ** for *p* < 0.01).

*E. coli* biofilm distribution on PDMS, bare glass and poly(MeOEGMA) brush was observed in real-time in the PPFC under hydrodynamic conditions similar to those found in urinary catheters and stents. The area fraction covered by the biofilm was chosen as a representative measure of adhesion during biofilm growth (0.5, 2 and 24 h) and antibiotic treatment for an additional 8 h period (32 h) (Figure 1). Lower surface coverages were registered on the polymer brush when compared to PDMS and glass surfaces for all time points (*p* < 0.01). After 24 h, in the polymer brush, the surface coverage decreased by 60% on average compared to PDMS and glass (*p* < 0.0001). Regarding the ampicillin treatment, the biofilm-covered area increased slightly during the 8 h of exposure (Figure 1). It can be noted that for the PDMS the increase of the area fraction covered by *E. coli* was about 46%, while for glass and the polymer brush surfaces, the increase reached 15% and 31%, respectively. Nevertheless, it is important to note that, even after the antimicrobial treatment, the poly(MeOEGMA) brush was the most effective surface in reducing cell adhesion, with 50% and 65% fewer cells than PDMS and glass, respectively (*p* < 0.0001).

**Figure 2:**
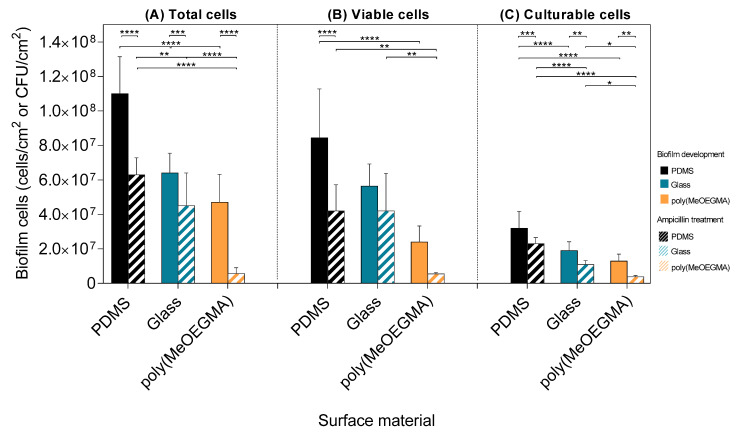
Number of (**A**) total, (**B**) viable, and (**C**) culturable cells after 24 h of biofilm development and after the ampicillin treatment on PDMS (■ and 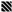, respectively), glass (■ and 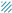, respectively), and poly(MeOEGMA) brush (■ and 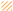, respectively). Standard deviations for three independent samples are presented. Statistical significance was determined by one-way ANOVA followed by Tukey’s multiple-comparison test (**** for *p* < 0.0001, *** for *p* < 0.001, ** for *p* < 0.01 and * for *p* < 0.05).

Figure 2 shows the number of total, viable and culturable cells on the tested surfaces before and after ampicillin exposure. The total (Figure 2A) and viable cell counts (Figure 2B) were determined by epifluorescence microscopy, whereas the biofilm culturable cells (Figure 2C) correspond to those that form colonies on agar-based media. Before treatment, PDMS had a higher number of total cells compared to glass and polymer brush (42% and 57% more cells, respectively; *p* < 0.0001, Figure 2A). The antibiotic treatment reduced the total cell amount on PDMS (43%) and glass (30%), but the most significant reduction was observed in the polymer brush (88%; *p* < 0.0001, Figure 2A). Even before the ampicillin treatment, the biofilm viability (i.e., the number of viable cells divided by the number of total cells) was 87% on average for the control surfaces and 52% for the polymer brush. The number of culturable cells (Figure 2C) was lower on glass and on the poly(MeOEGMA) brush when compared to PDMS surface, before and after antibiotic treatment (*p* < 0.0001, Figure 2C). Furthermore, the reduction of culturable cells after ampicillin treatment was about 28% on PDMS, 42% on glass and 71% on the polymer brush. 

**Figure 3:**
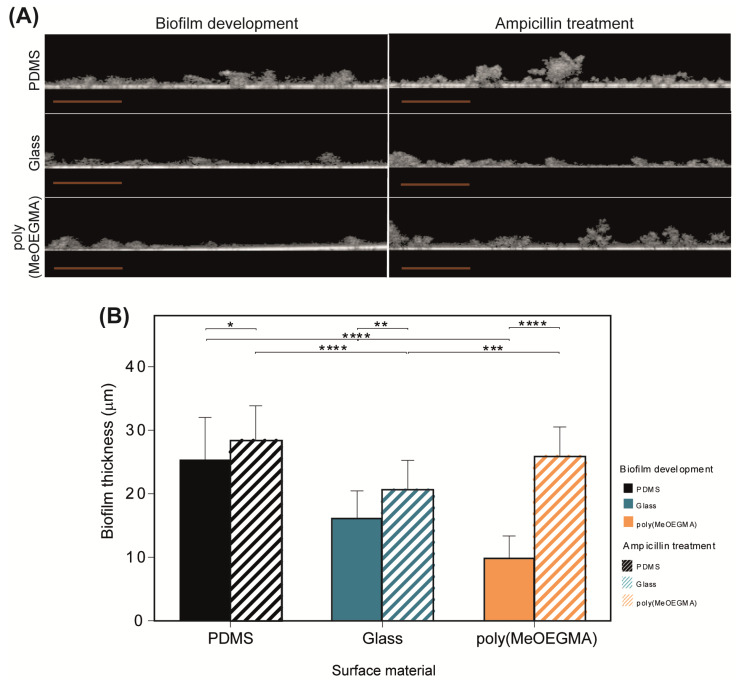
OCT analysis of biofilms formed on PDMS, glass and poly(MeOEGMA) brush: (**A**) representative 2D-cross-sectional views of biofilm structures (orange scale bars correspond to 200 µm), and (**B**) biofilm thickness after biofilm development and ampicillin treatment on PDMS (■ and 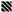, respectively), glass (■ and 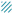, respectively) and poly(MeOEGMA) brush (■ and 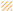, respectively). Standard deviations obtained from three replicates of each sample are represented. Statistical significance was determined by one-way ANOVA followed by Tukey’s multiple-comparison test (**** for *p* < 0.0001, *** for *p* < 0.001, ** for *p* < 0.01, and * for *p* < 0.05).

Optical coherence tomography (OCT) was the imaging technique selected to analyze the spatial distribution of biofilms developed on the surfaces and determine their thickness before and after antibiotic treatment (Figure 3). The two-dimensional (2D)-cross-sectional scans (Figure 3A) show the biofilm mass over the surface material represented by the white line. Figure 3B presents the average biofilm thickness values determined from the acquired 2D scans. The OCT cross-sectional views confirmed that after 24 h of biofilm development, the polymer brush showed a lower amount of biofilm compared to PDMS and glass (Figure 3A). Additionally, the representative OCT image of the biofilm formed on the polymer brush showed that this surface had less biofilm than the remaining surfaces, as previously revealed by brightfield microscopy (Figure 1). It is possible to conclude from Figure 3B that, prior to the ampicillin treatment, the biofilm formed on the poly(MeOEGMA) brush was thinner than those formed on PDMS and glass (61% and 40% thinner, respectively; *p* < 0.0001). However, after the antibiotic treatment, an increase in the biofilm amount was observed in all surfaces (Figure 3A) and higher thickness values were obtained compared to pre-treatment (11% higher for PDMS, 21% for glass and 62% for the polymer brush; *p* < 0.05, Figure 3B). The biofilm formed on the polymer brush seems to have suffered structural changes during the antibiotic treatment, acquiring the form of cell aggregates, which may have increased the biofilm thickness.

**Figure 4:**
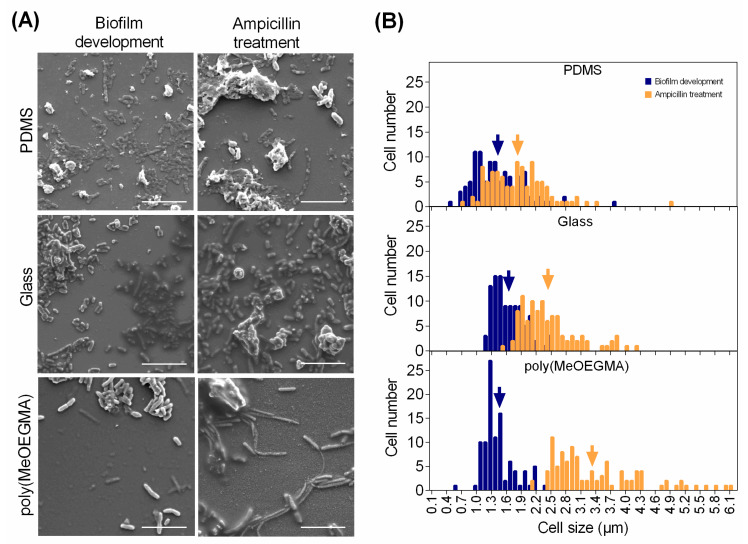
SEM analysis of biofilms formed after 24 h of development and after the ampicillin treatment on PDMS, glass and poly(MeOEGMA) brush: (**A**) representative micrographs (magnification: 5000×; bars = 10 µm); (**B**) cell size distribution of biofilms before (■) and after ampicillin exposure (■). The arrows represent the average cell length determined from SEM micrographs for each experimental condition.

SEM images showed that after biofilm growth, the number of adhered cells was higher on PDMS and glass than on the polymer brush (Figure 4A), which corroborates the quantification performed by epifluorescence microscopy (Figure 2A). Concerning the biofilms obtained after antibiotic exposure, higher surface coverages were observed on all tested surfaces (Figure 4A), particularly on the polymer brush, where some cell clusters were also visible, which supports the results obtained by brightfield microscopy (Figure 1) and OCT (Figure 3A). From SEM micrographs, it was also possible to determine the size distribution of biofilm cells on the three surfaces (Figure 4B). An average cell length of 1.5 µm was obtained for untreated biofilms, regardless of the surface material. However, after ampicillin exposure, the sessile cells on the polymer brush had an elongated morphology when compared to glass (on average 29% longer) and PDMS (on average 46% longer). Indeed, the *E. coli* cells that adhered to poly(MeOEGMA) brush were between 2.1 and 6.1 µm (57% longer than the poly(MeOEGMA) untreated cells), while the cells adhered to glass measured between 1.5 and 4.2 µm (30% longer than untreated cells obtained from glass) and the cells adhered to PDMS measured between 0.7 and 4.9 µm (17% longer than untreated cells obtained from PDMS).

## 3. Discussion

Several strategies have been developed to decrease bacterial adhesion and consequent biofilm formation on medical devices, including antiadhesive chemical surface modifications. The main goal of the current study was to evaluate the performance of the poly(MeOEGMA) brush on *E. coli* biofilm development and antibiotic susceptibility under flow conditions similar to those found in urinary catheters and ureteral stents where this bacterium has a significant impact. Biofilm development was followed for 24 h to more closely mimic a bladder infection with the continuous supply of bacteria. The biofilm growth was then monitored for an additional 8 h period where ampicillin was flown on the PPFC system to simulate the infection treatment with one of the most prescribed antibiotics for CAUTIs [41].

*E. coli* adhered more to PDMS and glass surfaces than to poly(MeOEGMA) after 24 h of biofilm development, revealing for the first time the long-term antifouling behavior of this polymer brush under urinary flow conditions. Resistance to fouling was expected from coatings with dense grafts, such as the poly(MeOEGMA) brush, which presents long polymer chains that are capable to minimize long-range interactions by creating an effective steric barrier which prevents the adhesion of macromolecules and cells to the surface [42,43]. Indeed, according to the extended Derjaguin–Landau–Verwey–Overbeek (DLVO) theory, attractive long-range interactions are reduced by introducing a water barrier with zero net charge and steric impediment in order to keep bacteria away from the surface [29]. The high wettability of the polymer brushes gives rise to an enthalpic barrier as water molecules have to be removed for proteins or bacteria to foul the surface [44]. Previous work from our research group [22] reported the strong antiadhesive effect of the polymer brush, with a reduction of 90% of cell adhesion in the first 30 min when citrate buffer was used as culture medium under the same hydrodynamic conditions. In the current work, synthetic urine medium was used to mimic the physiological conditions of the urinary system. An increase in adhesion on the polymer brush surface was evident when in contact with synthetic urine. This is probably related to the use of a high nutrient medium that may have formed a conditioning film, promoting bacterial adhesion [20,45]. Surface plasma resonance showed the resistance of the poly(MeOEGMA) brush to the adsorption of proteins or other macromolecules present in human urine [22]. After 15 min, a wavelength shift of less than 15 ng/cm^2^ was observed and upon 2 h of contact with urine, the fouling corresponded to less than 5% of the surface saturation [22]. This suggests that the antifouling properties of the polymer brush under the specific conditions of this work were probably surpassed by the intrinsic characteristics of *E. coli*, a motile organism containing bacterial appendages which may facilitate adhesion [46,47]. Concerning the susceptibility assays, the antibiotic ampicillin was able to remove cells from the biofilms on all of the tested surfaces, and the removal was higher with the polymer brush. The same effect of ampicillin was previously observed in 24-h *E. coli* biofilms formed on glass [48] where more than 50% of the sessile cells were removed after only 3 h of antibiotic exposure. The higher cell detachment from the polymer brush may be related to its weaker interaction with the bacterium [29,49]. According to Rodríguez-Emmenegger et al. [29], the work necessary to detach *Y. pseudotuberculosis* (a Gram-negative bacterium as *E. coli*) from brushes such as poly(MeOEGMA) and poly(HPMA) was between 2% and 20% of the work employed on glass. Besides its effect on cell removal, the antibiotic circulating into the PPFC system also promoted a higher reduction in the number of culturable cells of poly(MeOEGMA) brush compared to PDMS and glass surfaces.

The OCT images obtained after antibiotic treatment revealed that the biofilm cells adhered to the polymer brush experienced spatial reorganization, giving rise to aggregates that may be responsible for increasing the biofilm thickness. SEM analysis revealed that ampicillin-treated cells were more elongated than the untreated cells on all tested surfaces and formed cell clusters on the polymer brush. In addition, the elongation phenomenon was more noticeable on the brush, indicating a higher effect of the antibiotic treatment on biofilm cells. Previous studies have shown that *E. coli* cells in contact with ampicillin, a β-lactam antibiotic which acts to inhibit cell wall synthesis, may suffer morphological changes and spatial rearrangement [12,48,50,51]. These alterations may occur in four stages, including elongation, bulge formation, bulge stagnation and lysis [50]. The cluster formation and consequent increase in the biofilm thickness may be due to the overproduction of extracellular polymeric substances (EPS) in response to the osmotic stress resulting from cell–antibiotic interactions. Ionescu and Belkin [52] studied the *rpoS* function on *E. coli* strains and their findings suggest that the stress resistance is influenced by the presence of specific genes responsible for the EPS production. It has been reported that *rpoS* plays a major role in the osmotic stress response of *E. coli* by activating the transcription of a high number of genes that provide osmo- and cross-protection against different stress factors [53,54]. For *Enterococcus faecalis* (a bacterium responsible for CAUTIs as *E. coli* and typically susceptible to ampicillin), the formation of a complex 3D-biofilm structure in presence of antibiotics was also associated with a stress response of enterococcal biofilm cells, which promoted a rapid reorganization of biofilm into highly structured microcolonies [55]. A recent work evaluated the effects of ampicillin on surface modifications of four multidrug-resistant *E. coli* strains and concluded that three of them suffered cell elongation, an increase in roughness and nanoscale adhesion forces, became more hydrophilic and increased biofilm formation [56]. 

Overall, our results show that this polymer brush has potential advantages in the prevention of *E. coli* adhesion and biofilm formation in urinary tract devices, enabling a more effective eradication of biofilms when combined with antibiotic treatment, thus reducing the risk of recurrent infections.

## 4. Materials and Methods 

### 4.1. Test Organism and Culture Conditions

*E. coli* JM109(DE3) from Promega (Madison, WI, USA) was selected for its good biofilm-forming capacity in the same platform [14,22,49] and similar biofilm-forming capacity to different *E. coli* clinical isolates [40]. An overnight culture was prepared by inoculating 500 µL of a glycerol stock (kept at −80 °C) in a total volume of 0.2 L of synthetic urine medium. The medium was prepared as previously described [57] using the following formulation for 1 L of distilled water: peptone 1 g, yeast extract 0.05 g, lactic acid 1.1 mmol/L, citric acid 0.4 g, sodium bicarbonate 2.1 g, urea 10 g, uric acid 0.07 g, creatinine 0.8 g, calcium chloride.2H_2_O 0.37 g, sodium chloride 5.2 g, iron II sulphate.7H_2_O 0.0012 g, magnesium sulphate.7H_2_O 0.49 g, sodium sulphate.10H_2_O 3.2 g, potassium dihydrogen phosphate 0.95 g, di-potassium hydrogen phosphate 1.2 g and ammonium chloride 1.3 g. The pH of the solution was then adjusted to 6.5. All media components were purchased from Merck (Germany). The culture was grown on a 1 L shake-flask at 37 °C for 15–17 h with orbital agitation (160 rpm; IKA KS 130 basic, Staufen, Germany). Cells were harvested by centrifugation for 10 min at 3202× *g*. The suspension was then adjusted with synthetic urine medium to have an optical density at 610 nm of 0.1, equivalent to a cell density of 7.6 × 10^7^ cells/mL.

### 4.2. Surface Preparation 

Bare glass microscope slides (VWR, Portugal) and polydimethylsiloxane (PDMS) (Sylgard 184, Dow Corning, USA) were used as control surfaces. Glass was the substrate where the poly(MeOEGMA) brushes were grafted as in the previous studies addressing bacterial adhesion [22]. Glass was selected due to its transparency, which facilitates microscopy. PDMS is one of the most widely used polymers in the fabrication of medical implants for the urinary tract [58]. PDMS was prepared as previously described [14] and the poly(MeOEGMA) brushes were prepared according to Lopez-Mila et al. [22]. After preparation, round coupons of the poly(MeOEGMA) brush, PDMS and glass were sterilized by spraying with absolute ethanol (100%; Merck, Germany) for 5 min. Then, they were left to air-dry inside the laminar flow chamber and fixed to the bottom plate of the PPFC [22]. 

### 4.3. Biofilm Assays 

The bacterial suspension was recirculated through the PPFC system at 2 mL/s, which corresponds to the shear rate of 15/s found in urinary catheters [59]. As determined by computational fluid dynamics (CFD), the corresponding shear stress was 0.01 Pa [14] and previous studies revealed that this shear stress value can also be found in some problematic sites of ureteral stents that are prone to bacterial accumulation and encrustation [35,36]. Biofilm development at 37 °C was monitored in real-time with microscopic images acquired using the Nikon Eclipse LV100 microscope connected to a camera (Nikon digital sight DS-Ri 1, Japan) at different time points (0.5, 2 and 24 h). As it has been reported that *E. coli* biofilms in urinary catheters are completely mature in 24 h [7], after that period the *E. coli* suspension was removed from the system and pre-warmed, sterile, synthetic urine to a concentration equivalent to 5 × MBIC (minimal biofilm inhibitory concentration) of ampicillin (250 μg/mL, diluted in synthetic urine) [48] was flown on the system for 8 h at the same shear rate. Ampicillin (AppliChem, Germany) was used in the present work since it is an antibiotic commonly prescribed to treat CAUTIs [41]. Brightfield microscopy images were also acquired after the ampicillin treatment (the 32 h time point). The percentage of surface area covered by *E. coli* cells was calculated with the ImageJ software (version 1.38e; National Institutes of Health, EUA) for the 0.5, 2, 24 and 32 h time points as previously described [49].

### 4.4. Offline Quantification of Biofilm Cells 

Biofilms were developed for 24 h and at the end of each experiment the system was rinsed with synthetic urine to remove loosely attached cells. The flow cell was opened and the biofilm from each coupon was detached through the swabbing method [60,61] and a biofilm cell suspension diluted in 0.85% NaCl was obtained. To assess biofilm culturability (colony forming units, CFU), the biofilm suspension was properly diluted and spread on plate count agar (PCA, Oxoid, England). Colony enumeration was carried out after overnight incubation at 37 °C and the final values were expressed as CFU/cm^2^. The adhered cells were stained with 4´-6-diamidino-2-phenylindole (DAPI), which stains both viable and nonviable cells [48]. The viability of biofilm cells was determined with the Live/Dead^®^ (L/D) *Bac*Light^TM^ Bacterial Viability kit (Invitrogen Life Technologies, Alfagene, Portugal) as previously indicated [48]. All the samples were examined before (the 24 h time point) and after antibiotic exposure (the 32 h time point), in which a minimum of 20 fields of view of each stained sample was analyzed using the ImageJ software to estimate the total and viable cell numbers (expressed as cells/cm^2^). 

### 4.5. Optical Coherence Tomography (OCT)

In situ images of the biofilm structure were obtained with a Spectral Domain Optical Coherence Tomography (SD-OCT) system (Thorlabs GmbH, Dachau, Germany) with a central wavelength of 930 nm. The refractive index was set to 1.40, close to the refractive index of water (1.33), since water is the major component of biofilms [62]. Two-dimensional (2D) images were acquired after 24 h of biofilm formation and after the ampicillin treatment (the 32 h time point). The biofilm thickness profiles were determined in at least five images corresponding to different locations for each surface at different time points. For each image, a minimum of 25 points was analyzed using the Thorlabs software tool. 

### 4.6. Scanning Electron Microscopy (SEM)

The morphology of *E. coli* biofilms formed after 24 h and after exposure to ampicillin was analyzed by SEM. Coupons were removed from the PPFC and gradually dehydrated with ethanol at different concentrations (10, 25, 40, 50, 70, 80, 90 and 100% *v*/*v*) [63]. The samples were then air-dried and sputter-coated with a palladium-gold thin film [12]. The biofilms were observed with an SEM/EDS system (FEI Quanta 400FEG ESEM/EDAX Genesis X4M, FEI Company, USA) in high-vacuum mode at 15 kV. Cell length was determined for each condition as described by Gomes and Mergulhão [12].

### 4.7. Statistical Analysis

Experiments were carried out in triplicate using different *E. coli* overnight cultures. Graph production and statistical analysis were performed using GraphPad Prism 6.01 (La Jolla, USA). Data were presented as mean ± standard deviations. Statistical differences between the polymer brush and the control surfaces (PDMS and glass) were determined using one-way analysis of variance (ANOVA) followed by Tukey’s multiple-comparison test.

## 5. Conclusions

Biofilms causing CAUTIs resist antibiotic treatment and often require replacement of infected devices. Current strategies for developing infection-resistant biomaterials have gone through making them non-fouling, bactericidal or both. However, there are few studies that evaluate these new materials in conditions that mimic the real environment where they will be applied. In this study, special attention was paid to hydrodynamic and nutrient conditions since we believe these are critical parameters in translating research into practical applications. The antifouling performance that the poly(MeOEGMA) brush demonstrated in a previous work with citrate buffer was maintained here for a longer period of time. The ability of the polymer brush to significantly delay *E. coli* biofilm formation when compared to PDMS (a material commonly used in the manufacture of medical settings) makes it a very promising surface to prevent infections in urinary tract devices like urinary catheters and ureteral stents. This study also suggests that the further grafting of active antimicrobial agents such as antibiotics onto the surface of poly(MeOEGMA) brush may favor the detachment of pre-formed biofilms due to the weakly adhering cells on the polymer brush.
